# O2-5 Mobilising People as Assets for community-based active ageing promotion: A multi-stakeholder perspective on peer volunteering initiatives

**DOI:** 10.1093/eurpub/ckac094.013

**Published:** 2022-08-29

**Authors:** Afroditi Stathi, Janet Withall, Sandra Agyapong-Badu, Eva Barrett, Marlene Kritz, Cecilie Thogersen-Ntoumani, Isobel Whitaker, Harry Toon, Kenneth Fox

**Affiliations:** 1 School of Sport, Exercise and Rehabilitation Sciences, University of Birmingham, Birmingham, United Kingdom; 2 Department for Health, University of Bath, Bath, United Kingdom; 3 School of Nursing & Midwifery, National University of Ireland, Galway, Ireland; 4 School of Psychology, Curtin University, Perth, Australia, Austria; 5 School of Policy Studies, University of Bristol, Bristol, United Kingdom

**Keywords:** community assets, peer volunteering, peer support, physical activity, active ageing

## Abstract

**Background:**

Citizens who contribute as volunteers and peer mentors within a community are important assets that can be mobilised to improve health and wellbeing. In order to optimise the contribution of peer-volunteers to active ageing initiatives, we need to understand their experiences and identify ways to support them in preparing for the role and overcoming potential challenges.

Aim: This study synthesises the perspectives of a range of stakeholders involved in peer volunteering active ageing initiatives implemented in UK and provides an in-depth account of how such initiatives can effectively mobilise individuals as community assets. It draws on the experience of all actors involved in such initiatives, ranging from volunteers, recipients of volunteering actions, volunteer managers and volunteering service providers.

**Methods:**

This evidence synthesis used qualitative data from (a) three studies focusing on the development (phase 1), feasibility trial evaluation (phase 2) and community roll-out (phase 3) of ACE (Active, Connected, Engaged), a peer volunteering active ageing intervention, alongside (b) the experiences of third sector organisations in providing peer volunteering programmes. Ten managers, 22 volunteers and 20 ACE participants were interviewed. Interviews were audio-recorded, transcribed verbatim and analysed using framework analysis.

**Results:**

Seven main themes including 33 higher and 22 lower order themes were identified: Motives; Benefits; Characteristics of peer volunteers; Challenges; Training needs; Recruitment; Successful strategies for maintenance. Altruism, changes in life circumstances, opportunities to reconnect with the community and personal fulfilment were the main reasons for volunteering. Volunteering was described as being personally rewarding, an avenue to acquire new skills and knowledge, and increased social connections and physical activity. Effective volunteers are committed, reliable, have a good sense of humour, good interpersonal skills and are able to relate to the participants. When pairing volunteers with participants, clarity of role, level of time commitment, shared interests and geographical proximity are worth considering.

**Conclusions:**

Successful implementation of peer-volunteering initiatives requires the use of appropriate recruitment routes, easy joining procedures, provision of ongoing support, good communication across all stakeholders, feedback and recognition of effort and a dedicated team to build resilience and provide volunteers with administrative support.

